# Routine Versus Clinically Indicated Replacement of Peripheral Intravenous Catheters in Adults: A Non-Inferiority Cluster-Randomised Crossover Trial

**DOI:** 10.3390/healthcare14131825

**Published:** 2026-06-23

**Authors:** Nuri Kang, Hyun Lim Kim, Hye Ran Choi, Jeounghee Kim

**Affiliations:** 1 Department of Nursing, Asan Medical Center, Seoul 05505, Republic of Korea; nurikang@amc.seoul.kr (N.K.); carpediem@amc.seoul.kr (H.L.K.); 2 Department of Clinical Nursing, University of Ulsan College of Medicine, Seoul 05505, Republic of Korea; hr.choi@amc.seoul.kr

**Keywords:** peripheral intravenous catheter, phlebitis, clinically indicated replacement, non-inferiority trial, cluster-randomised crossover clinical trial, Infection control, nursing practice

## Abstract

**Highlights:**

**What are the main findings?**

- Clinically indicated catheter replacement was non-inferior to routine 96-h replacement for phlebitis.
- No catheter-related bloodstream infections were observed in either group, and clinically indicated replacement required fewer catheter insertions per patient.

**What are the implications of the main findings?**

- Clinically indicated replacement may reduce unnecessary catheter insertions while maintaining patient safety.
- These findings support a less invasive and potentially more efficient PIVC replacement strategy in adult inpatients.

**Abstract:**

**Background/Objectives**: Routine replacement of peripheral intravenous catheters (PIVCs) every 72–96 h remains common practice despite growing guideline support for clinically indicated replacement. Evidence from Asian healthcare settings remains limited. **Methods**: A non-inferiority, cluster-randomised crossover trial was conducted from January to July 2021 across eight wards of a tertiary care hospital in South Korea. Adults requiring peripheral IV therapy for ≥96 h were allocated to routine 96-h replacement or clinically indicated replacement. The primary outcome was phlebitis incidence; a non-inferiority margin of 5% (absolute risk difference) was prespecified. **Results**: Among 1324 participants, phlebitis occurred in 12.6% (clinically indicated) vs. 11.7% (routine) (ARD 1.44 pp, 95% CI −1.47 to 4.35), meeting non-inferiority. No catheter-related bloodstream infections were observed in either group. The clinically indicated group required fewer catheter insertions (mean 1.77 vs. 2.16; *p* < 0.001) and had longer dwell times (mean 112.0 vs. 89.6 h; *p* < 0.001). **Conclusions**: Clinically indicated PIVC replacement was non-inferior to routine scheduled replacement for phlebitis and was associated with fewer insertions and longer dwell times, supporting its use in routine clinical practice.

## 1. Introduction

Peripheral intravenous (IV) catheterisation is one of the most frequently performed procedures for administering fluids and medications to hospitalised patients. Despite its routine nature, peripheral intravenous catheter (PIVC) use is associated with complications such as phlebitis, infiltration, pain, occlusion, and catheter-related bloodstream infection (CRBSI), which may compromise patient safety and increase healthcare utilisation [1,2]. Phlebitis is the most common complication associated with PIVC and is characterised by signs and symptoms such as pain, erythema, swelling, warmth, and tenderness at the catheter site [3]. Because phlebitis has traditionally been considered a major catheter-related complication, routine replacement of PIVCs every 72–96 h was widely adopted to reduce its occurrence and improve patient safety [4].

The 2011 guidelines from the Centers for Disease Control and Prevention (CDC) recommend against routine replacement of PIVCs more frequently than every 72–96 h and describe clinically indicated replacement as an unresolved issue, citing limited evidence at that time [4]. Clinically indicated replacement refers to catheter removal only when a clinical reason is identified, such as phlebitis, infiltration, occlusion, suspected infection, patient discomfort, or completion of therapy [3]. Subsequent international studies comparing routine and clinically indicated replacement have reported inconsistent findings, particularly with respect to phlebitis and CRBSI incidence, resulting in ongoing uncertainty regarding optimal catheter replacement strategies [4,5,6].

In response to the evolving evidence base, several guideline bodies have updated their recommendations to support clinically indicated catheter replacement [7,8], including the Infusion Nurses Society (INS), whose position is reflected in the 2024 Infusion Therapy Standards of Practice [3], and the National Institute for Health and Care Excellence (NICE). In Korea, similar recommendations were adopted by the Korean Hospital Nurses Association in 2017 [9]. However, the direct applicability of evidence supporting clinically indicated replacement to settings with different patient populations and vascular access practices remains an important consideration. Although a small number of randomised trials have been conducted in Asian settings, including China, large-scale randomised evidence from Korean clinical practice remains scarce, and no prior cluster-randomised trial has evaluated clinically indicated PIVC replacement with dwell times extending beyond 96 h in this context [2,10]. PIVC-related complications may be influenced by patient characteristics, catheter-related factors, and institutional practices [3,5]. Therefore, additional randomised evidence from clinical settings that have been less extensively evaluated in previous trials can strengthen the generalisability of clinically indicated replacement strategies.

Routine catheter replacement places a substantial burden on nursing workload and has been highlighted in initiatives such as the Choosing Wisely campaign as a potentially low-value practice in the absence of clear clinical benefit [11]. Clinically indicated replacement has been proposed as an alternative approach that may reduce patient discomfort, healthcare costs, and nursing workload while maintaining safety [8,12,13]. Nevertheless, concerns remain regarding prolonged catheter dwell times and their potential association with catheter-related complications [14,15,16].

To address this evidence gap, we conducted a non-inferiority, cluster-randomised crossover trial to evaluate the safety of clinically indicated compared with routine PIVC replacement in a Korean tertiary care hospital.

## 2. Methods

### 2.1. Study Design and Setting

**Figure 1:**
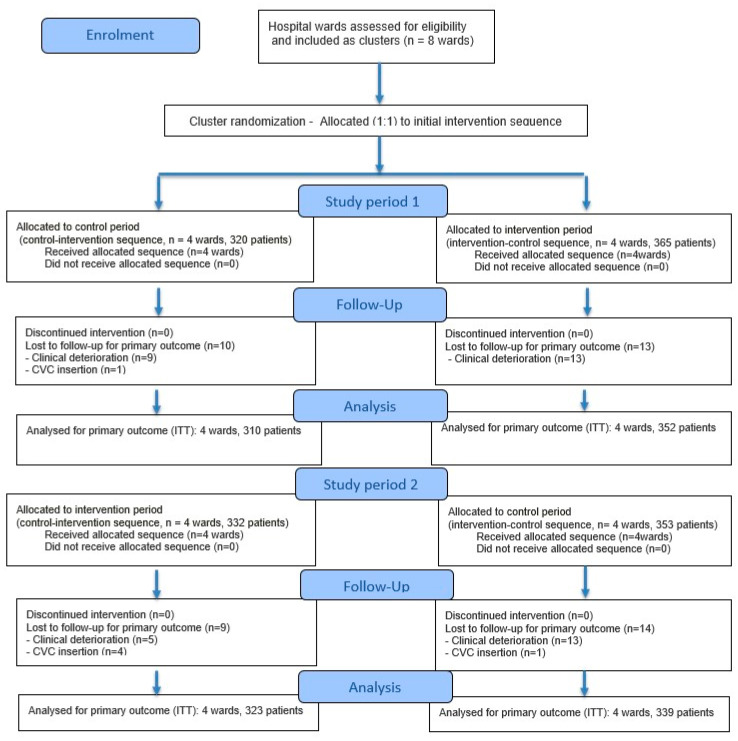
CONSORT flow diagram of participant enrollment and analysis. Flow diagram of the progress of clusters and patients through the phases of a cluster-randomised crossover trial (enrollment, intervention allocation, follow-up, and analysis). A total of 1324 participants (675 in the clinically indicated group and 649 in the routine replacement group) were included in the intention-to-treat analysis. Abbreviations: ITT, intention to treat; CVC, central venous catheter.

We conducted an open-label, non-inferiority, cluster-randomised crossover (CRXO) clinical trial to compare routine 96-h peripheral intravenous (IV) catheter replacement with clinically indicated replacement in adult inpatients. The trial was performed at a 2700-bed tertiary academic hospital in Seoul, South Korea. Eight wards (four medical and four surgical) were randomised to one of two intervention sequences using stratified block randomization according to ward type. The random allocation sequence was generated by an independent statistician from the institutional biostatistics team using a computer-generated randomization program. The allocation sequence was not accessible to investigators prior to assignment. Due to the nature of the intervention, neither participants nor investigators could be blinded to group allocation. After the first study period, wards crossed over to the alternate intervention according to the predefined crossover design (Figure 1).

In the clinically indicated group, catheters were replaced only when signs of complications emerged, including phlebitis, infiltration, suspected catheter-related bloodstream infection (CRBSI), or patient discomfort. In both groups, catheters were removed under identical clinical conditions irrespective of the scheduled replacement interval. In cases of suspected CRBSI, catheter tips were aseptically collected for microbiological culture.

### 2.2. Participants and Sampling

Eligible participants were adults aged ≥19 years who were admitted to the study wards and required peripheral IV therapy for at least 96 h. Exclusion criteria included pre-existing bloodstream infection, use of immunosuppressive agents, active skin or inflammatory conditions at the insertion site, and concurrent central venous catheterisation. Participants were withdrawn if exclusion criteria were met after enrolment or if consent was withdrawn.

Sample size was calculated using PASS software (version 15.0.7) for a non-inferiority design, based on an expected phlebitis incidence of 11.3% in both groups, a non-inferiority margin of 5%, a one-sided significance level of 0.025, and a statistical power of 80%. The non-inferiority margin of 5% (absolute risk difference in phlebitis incidence) was prespecified on the basis of both clinical judgment and precedent from prior randomised trials. Rickard et al. (2012) employed a 3% absolute margin in a large equivalence trial of routine versus clinically indicated PIVC replacement [17], and subsequent trials and the Cochrane review by Webster et al. (2019) have accepted margins ranging from 3% to 5% as clinically acceptable [8], given the self-limiting nature of most phlebitis events and their low associated morbidity. A 5% margin was considered acceptable in the present context because the potential benefits of clinically indicated replacement—reduced patient discomfort from repeated cannulation, lower nursing workload, and decreased resource utilization —would, in the judgment of the investigators and participating clinicians, outweigh a small absolute increase in low-grade phlebitis risk within this range. Given that the study involved ward-level allocation, an intraclass correlation coefficient (ICC) of 0.02 was assumed to account for potential clustering effects. The initial calculation indicated that 615 participants per group were required, resulting in a total sample size of 1230 participants. Considering a potential withdrawal rate of 10%, the final sample size was increased to 1370 participants.

### 2.3. Outcomes and Measurements

The primary outcome was the incidence of phlebitis, assessed using the Infusion Nurses Society (INS) phlebitis scale, which grades severity from 0 (no symptoms) to 4 (pain with erythema, a palpable venous cord >1 cm, and purulent drainage) [18].

Secondary outcomes included the incidence of CRBSI, defined according to Centers for Disease Control and Prevention (CDC) criteria as the isolation of the same organism from both the catheter tip and a peripheral blood culture, accompanied by clinical signs of infection and no alternative source [4]. Infiltration or extravasation was assessed using the INS infiltration scale (grades 0–4), which evaluates severity based on oedema size, skin changes, and pain [18]. Infusion failure was defined as premature catheter removal due to occlusion, discomfort unrelated to other complications, accidental removal, or leakage.

Additional secondary outcomes included the mean number of catheter insertions per patient, catheter dwell time, and total duration of intravenous therapy.

### 2.4. Procedures

The study was conducted from 18 January to 9 July 2021 across two intervention periods. A dedicated IV team consisting of 35 trained nurses performed most catheter insertions. Both ward nurses and IV team members completed standardised IV therapy training, including phlebitis assessment and documentation procedures, before study initiation.

All PIVC insertions followed a standard aseptic protocol, including hand hygiene using 70% ethanol, skin disinfection with 83% alcohol, and insertion of polyurethane-based PIVCs. Transparent polyurethane dressings were used and replaced as needed or every seven days. Needleless connectors were changed only when visibly contaminated or functionally compromised.

Each catheter was tracked using a case report form. Catheter-specific data were recorded by the inserting nurse, and catheter removal data were extracted from electronic medical records by IV team members.

### 2.5. Data Analysis

All statistical analyses were performed using SAS software version 9.4 (SAS Institute, Cary, NC, USA). Categorical variables were summarised as frequencies and percentages, and continuous variables as means with standard deviations. Group comparisons were conducted using the chi-square test for categorical variables and the independent *t*-test for continuous variables. Analyses were performed for both the intention-to-treat (ITT) and per-protocol (PP) populations.

To account for the cluster-randomised crossover design, ward was treated as the unit of clustering in all inferential analyses. For the primary outcome, relative risks (RRs) and absolute risk differences (ARDs) with 95% confidence intervals (CIs) were estimated using generalized estimating equation (GEE) models with an exchangeable working correlation structure and robust (sandwich) standard errors, with ward specified as the clustering variable and study period included as a fixed effect.

Time-to-event analyses were conducted using Cox proportional hazards models stratified by study period. Cluster-robust standard errors were obtained using the robust sandwich variance estimator (COVS(AGGREGATE) option in SAS PROC PHREG), with ward specified as the clustering variable. The proportional hazards assumption was assessed graphically using log–log survival plots and formally tested using Schoenfeld residuals.

Incidence rates per 1000 catheter-days were calculated based on the first occurrence of phlebitis per patient, and corresponding 95% CIs were estimated using exact Poisson methods. Secondary outcomes were analysed using Cox proportional hazards models with cluster-robust variance estimation, consistent with the time-to-event nature of these outcomes.

Non-inferiority was concluded if the upper bound of the 95% CI for the ARD was below the prespecified margin of 5%. All statistical tests were two-sided, and a *p*-value < 0.05 was considered statistically significant. Missing data were handled using complete case analysis. No important changes to the study methods or outcomes were made after trial commencement.

### 2.6. Ethical Considerations

This cluster-randomised crossover trial was conducted in accordance with the principles of the Declaration of Helsinki and relevant national guidelines for research involving human participants. The study protocol was reviewed and approved by the Institutional Review Board of Asan Medical Center, Seoul, Republic of Korea (IRB approval no. 2020-1086; approval date 10 July 2020). The study was registered with the Clinical Research Information Service (KCT0005805). Both catheter replacement strategies evaluated in this trial (routine replacement every 96 h and clinically indicated replacement) were consistent with contemporary clinical practice and existing institutional policies and were considered to fall within the range of acceptable standard care.

Because the intervention was implemented at the ward (cluster) level and involved modification of routine catheter replacement policies within the scope of standard care, the Institutional Review Board granted a waiver of individual written informed consent. Patients (and, where appropriate, their families) received information about the study and the catheter replacement policies through routine clinical communication, and were allowed to decline the study procedures or request catheter replacement at any time without any impact on the quality of their usual care. All data were obtained from existing medical records, de-identified before analysis, and stored on secure, password-protected servers accessible only to the research team.

## 3. Results

### 3.1. Participant Characteristics

Participant flow through the study is shown in Figure 1. A total of 1324 participants were included in the intention-to-treat (ITT) analysis, with 675 in the clinically indicated group and 649 in the routine replacement group. The two groups were comparable with respect to sex, age, and department type (Table 1). Most peripheral IV catheters were inserted by the dedicated IV team, accounting for more than 92% of insertions in both groups.

A significant difference was observed in catheter gauge distribution (χ^2^ = 5.90, *p* < 0.001), with a higher proportion of 24-gauge catheters used in the clinically indicated group (Table 2). No significant differences were identified in catheter insertion site or baseline phlebitis grade.

### 3.2. Primary Outcomes

In the intention-to-treat (ITT) population, phlebitis occurred in 12.6% of participants in the clinically indicated group and 11.7% in the routine replacement group. When accounting for the cluster-randomised crossover design using generalized estimating equation models, the relative risk (RR) was 1.13 (95% confidence interval [CI] 0.90–1.41; *p* = 0.301). The absolute risk difference (ARD) was 1.44 percentage points (95% CI −1.47 to 4.35), and the upper bound of the confidence interval remained below the prespecified non-inferiority margin of 5%, confirming non-inferiority (Table 3).

**Figure 2:**
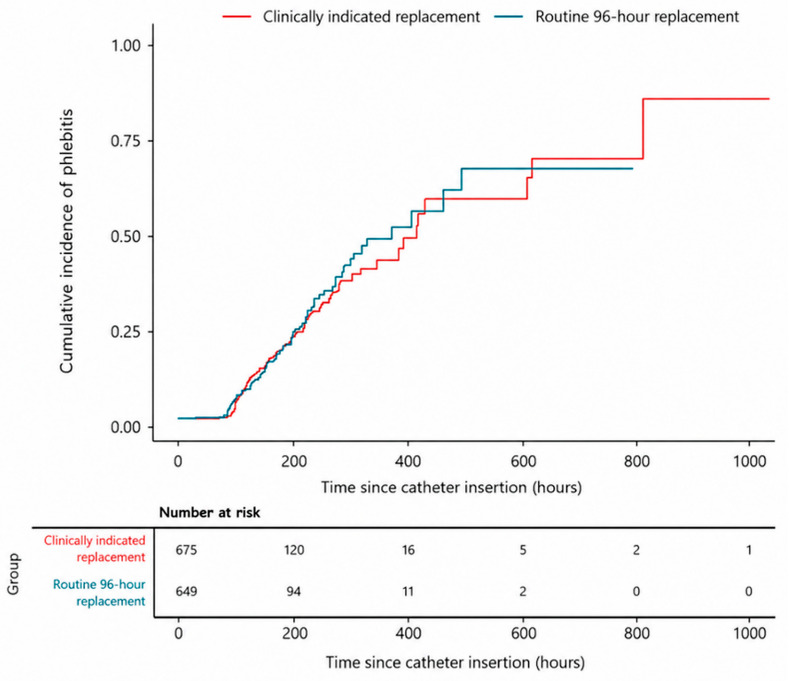
Kaplan–Meier analysis of survival from phlebitis per patient. Kaplan–Meier curves illustrate time to first occurrence of phlebitis in the clinically indicated replacement group and the routine replacement group.

Similar findings were observed in the per-protocol analysis (ARD 1.50 percentage points, 95% CI −1.18 to 4.18; *p* = 0.272), supporting the robustness of the primary result. The incidence rate of phlebitis per 1000 catheter-days was 19.96 (95% CI 15.94–24.68) in the clinically indicated group and 19.34 (95% CI 15.24–24.20) in the routine replacement group, indicating no meaningful difference between groups (Table 3; Figure 2).

Time-to-event analysis using Kaplan–Meier curves demonstrated broadly overlapping survival-from-phlebitis functions in the two groups throughout the observation period (Figure 2). The two curves remained closely aligned during the first 200 h following catheter insertion, with only minor divergence observed at later time points as a greater proportion of catheters in the clinically indicated group remained in situ beyond the conventional 96-h threshold. The number of catheters at risk decreased progressively over time in both groups, consistent with the expected attrition of PIVC observations. The log-rank test revealed no statistically significant difference between the two survival distributions (*p* = 0.716), and the hazard ratio for phlebitis derived from a Cox proportional hazards model stratified by study period was 1.06 (95% CI 0.81–1.38; *p* = 0.675), consistent with the primary non-inferiority finding reported above.

### 3.3. Secondary Outcomes

No catheter-related bloodstream infection events were reported in either group. The incidence of infiltration or extravasation, occlusion, discomfort, accidental removal, and leakage did not differ significantly between groups (all *p* > 0.05) (Table 3).

Regarding resource utilisation, the clinically indicated group required significantly fewer catheter insertions per patient than the routine replacement group (mean 1.77 vs. 2.16; median [range], 1 [1–14] vs. 2 [1–10]; *p* < 0.001) and demonstrated a significantly longer mean catheter dwell time (mean 112.0 vs. 89.6 h; median [range], 104.0 [5.4–350.0] vs. 89.1 [14.3–120.4] h; *p* < 0.001) (Table 4). No significant difference was observed in the total duration of intravenous therapy between groups (*p* = 0.910).

## 4. Discussion

This study demonstrated that clinically indicated peripheral intravenous (IV) catheter replacement was non-inferior to routine 96-h replacement with respect to phlebitis incidence. The absolute risk of 1.44% (95% CI −1.47 to 4.35) remained below the prespecified non-inferiority margin, and findings were consistent across both intention-to-treat and per-protocol analyses. These results are consistent with previous randomised trials and with a recent systematic review and meta-analysis supporting clinically indicated catheter replacement strategies [2,5,15,16].

The observed phlebitis rates in both groups (11.7–12.6%) exceeded the <5% benchmark suggested by the Infusion Nurses Society [3], but were comparable to rates reported in other recent clinical studies, including a large multicentre Spanish cohort [10,12,16,19]. Variability across studies likely reflects differences in clinical environments and practices, including skin antisepsis protocols, dressing materials, and broader healthcare system factors [5,12,15,16,17,18].

Notably, the frequent use of larger-bore catheters (18 G–20 G) in our setting, which exceeded that reported in previous studies [5,12,15], may have contributed to the relatively higher phlebitis rates observed. Prior evidence supports the use of the smallest catheter gauge appropriate to clinical need to minimise endothelial irritation [3,5]. Periodic reassessment of catheter gauge following surgery or invasive procedures may therefore be important to reduce phlebitis risk.

No Grade 4 phlebitis or CRBSI events were observed in either group. The absence of CRBSI is consistent with previous evidence indicating that bloodstream infection related to PIVCs is rare [8,20]. However, phlebitis and CRBSI are distinct clinical outcomes; therefore, phlebitis findings should not be used to infer bloodstream infection risk. Because this trial was not powered to evaluate rare infectious outcomes and no CRBSI events occurred, the present study cannot determine whether bloodstream infection risk differed between replacement strategies. Larger multicentre trials or surveillance-based investigations are needed to evaluate the effect of clinically indicated replacement on CRBSI risk.

Clinically indicated replacement was associated with fewer catheter insertions per patient and longer catheter dwell times, indicating potential reductions in patient discomfort and procedural burden while maintaining safety. These findings support the potential for more resource-efficient care without compromising clinical outcomes [7]. Previous studies suggest that most PIVC-related complications, including phlebitis, occur within the first 96 h after insertion [21], whereas systemic infections such as CRBSIs remain uncommon [8]. Nevertheless, sustained monitoring and early recognition of complications remain essential, particularly in routine clinical practice where surveillance intensity may vary. The Kaplan–Meier analysis provides clinically meaningful information that complements the primary non-inferiority finding. The closely overlapping survival-from-phlebitis curves throughout the observation period (Figure 2) indicate that the risk of phlebitis did not accumulate differently between the two replacement strategies over time, even as catheter dwell times extended well beyond the conventional 96-h threshold in the clinically indicated group. This pattern suggests that scheduled 96-h replacement does not confer a time-dependent protective advantage against phlebitis in this population; rather, the timing of phlebitis onset appears to be driven primarily by patient- and catheter-level factors rather than by the replacement policy itself. From a practical standpoint, these findings imply that longer catheter dwell times under a clinically indicated strategy can be sustained without a measurable increase in phlebitis hazard, which translates into fewer cannulation events per patient, reduced patient discomfort from repeated venipuncture, and a meaningful reduction in nursing workload associated with routine catheter replacement—benefits that are consistent with the resource-use findings reported in Table 4.

Although specialised IV teams commonly focus on catheter insertion, post-insertion management plays a critical role in preventing complications. Evidence supports the use of structured approaches such as hand hygiene reinforcement, aseptic non-touch technique, and care bundles to optimise catheter maintenance and timely removal [22,23]. Development of locally tailored, evidence-based management frameworks may further enhance patient safety.

Debate continues regarding optimal catheter replacement strategies. Clinically indicated replacement offers advantages in terms of patient comfort, staff workload, and resource use, while concerns persist regarding rare but serious infections associated with prolonged dwell times [14]. Context-specific policy decisions should therefore balance safety considerations with operational efficiency and patient-centred outcomes.

### Limitations and Strengths

This study has several limitations. First, the trial was conducted at a single tertiary academic centre with a dedicated IV team, which may limit generalisability to community hospitals, long-term care facilities, or settings without specialised vascular access teams. Second, the open-label design may have introduced observer bias in phlebitis ascertainment; this was mitigated by standardised assessor training and use of the Infusion Nurses Society phlebitis scale. Although phlebitis and other catheter-related complications were assessed using predefined criteria and standardised evaluation tools, some clinical signs of local inflammation may be subject to variation in interpretation among assessors, and a degree of inter-observer variability cannot be completely excluded. Furthermore, while phlebitis is a well-characterised and straightforward clinical endpoint that can be reliably assessed at the bedside using validated grading tools, other manifestations of catheter-related infection or local inflammation—such as early-stage subcutaneous infection or systemic inflammatory responses—are considerably more difficult to characterise and grade consistently in routine clinical practice. The absence of a formal surveillance protocol specifically targeting these endpoints represents an additional limitation of the complication ascertainment in this trial. Third, the cluster-randomised crossover design, while pragmatic and efficient, may be affected by period effects, secular changes in clinical practice, or carryover of ward-level catheter-management behaviours between study periods. Analyses stratified by study period were performed to mitigate these effects; however, unmeasured cluster-period effects and limited precision due to the small number of clusters cannot be completely excluded. Finally, the trial was not powered for rare outcomes such as CRBSI, and the eligibility criteria (adult inpatients requiring ≥96 h of peripheral IV therapy) limit applicability to paediatric, immunocompromised, or outpatient populations.

Despite these limitations, the cluster-randomised crossover design reduced between-ward contamination and enabled pragmatic evaluation under routine clinical conditions. The main contribution of this study is that it adds randomised evidence from a Korean tertiary care setting to a literature largely derived from other healthcare contexts, thereby strengthening the generalisability of clinically indicated PIVC replacement. To our knowledge, this is the first randomised controlled trial of clinically indicated PIVC replacement with extended dwell times beyond 96 h conducted in a Korean tertiary care setting. The use of a dedicated IV team, a standardised aseptic protocol, and electronic medical record–based outcome ascertainment further strengthened the internal validity of the findings.

## 5. Conclusions

Clinically indicated PIVCs replacement was non-inferior to routine scheduled replacement with respect to phlebitis and was associated with fewer catheter insertions and longer catheter dwell times. Because no CRBSI events occurred in either group, this study could not determine whether clinically indicated replacement affected CRBSI risk. These findings support the feasibility of a clinically guided replacement strategy in a tertiary care setting, while highlighting the need for continued monitoring and further studies across diverse clinical environments. Implementation of standardised management protocols, including care bundles tailored to clinically indicated replacement, may help optimise patient outcomes and reduce IV catheter-related complications.

## Figures and Tables

**Table 1:** Baseline demographic and clinical characteristics of participants.

Characteristics	Category	Clinically Indicated (N = 675)	Routine Replacement (N = 649)	χ^2^/t	*p*
Gender	Male	412 (61.04%)	419 (64.56%)	1.16	0.246
	Female	263 (38.96%)	230 (35.44%)		
Age (years)		60.01 (12.00)	60.51 (12.57)	−0.49	0.623
Department	Medical	264 (39.11%)	280 (43.14%)	1.38	0.752
	Surgical	411 (60.89%)	369 (56.86%)		

Values are presented as mean ± standard deviation or number (%). *p*-values were calculated using the independent *t*-test for continuous variables and the chi-square test for categorical variables.

**Table 2:** Catheter-related characteristics at baseline.

Characteristics	Categories	Clinically Indicated (N = 1197)	Routine Replacement (N = 1415)	χ^2^/t	*p*
Inserted by	IV team	1107 (92.48%)	1306 (92.29%)	−0.14	0.889
	Staff nurse	90 (7.51%)	109 (7.70%)		
Catheter gauge	18 G	109 (9.11%)	172 (12.16%)	5.90	<0.001
	20 G	288 (24.06%)	452 (31.94%)		
	22 G	323 (26.89%)	361 (25.51%)		
	24 G	477 (39.85%)	430 (30.39%)		
Insertion side	Left side	586 (48.96%)	645 (45.58%)	−1.72	0.085
	Right side	611 (51.04%)	770 (54.42%)		
Insertion site	Hand	189 (15.79%)	203 (14.35%)	0.02	0.986
	Wrist	75 (6.27%)	83 (5.87%)		
	Forearm	827 (69.09%)	1045 (73.85%)		
	Cubital fossa	81 (6.77%)	58 (4.10%)		
	Upper arm	15 (1.25%)	9 (0.64%)		
	Others	10 (0.84%)	15 (1.06%)		
Severity of phlebitis	Grade 0	1085 (90.46%)	1310 (92.58%)	1.56	0.120
	Grade 1	82 (6.85%)	77 (5.44%)		
	Grade 2	25 (2.09%)	22 (1.55%)		
	Grade 3	5 (0.42%)	6 (0.42%)		
	Grade 4	0 (0%)	0 (0%)		

Values are presented as number (%). *p*-values were calculated using the chi-square test. Baseline phlebitis severity was assessed at the time of catheter insertion. All analyses in this table were conducted at the peripheral intravenous catheter (PIVC) level. Abbreviations: G, gauge; IV, intravenous.

**Table 3:** Study outcomes by treatment group.

	Clinically Indicated	Routine Replacement	Risk (95% CI)				*p*
	(*n* = 675)	(*n* = 649)		Estimate	Lower	Upper	
Primary outcome (ITT analysis, *n* = 1324)							
Phlebitis per patient, *n* (%)	85 (12.59%)	76 (11.71%)	RR	1.127	0.899	1.413	0.301
			ARD	1.44	−1.47	4.35	
Phlebitis per 1000 catheter days (95% CI)	19.96 (15.94–24.68)	19.34 (15.24–24.20)	HR	1.059	0.81	1.384	0.675
Primary outcome (PP analysis, *n* = 1285)							
Phlebitis per patient, *n* (%)	79 (12.04%)	69 (10.97%)	RR	1.141	0.915	1.422	0.241
			ARD	1.50	−1.18	4.18	
Phlebitis per 1000 catheter days (95% CI)	19.42 (15.37–24.20)	18.37 (14.29–23.25)	HR	1.057	0.798	1.401	0.699
Secondary outcomes (*n*, per 1000 catheter days)							
CRBSI	0 (0.0%)	0 (0.0%)	HR	-	-	-	-
Infiltration, Extravasation	115 (27.15%)	92 (23.58%)	HR	1.239	0.978	1.571	0.076
Occlusion	68 (16.05%)	45 (11.53%)	HR	1.318	0.934	1.860	0.117
Uncomfortable	112 (26.44%)	75 (19.22%)	HR	1.280	0.988	1.657	0.062
Accidental removal	17 (4.01%)	15 (3.84%)	HR	0.997	0.527	1.886	0.993
Leakage	16 (3.78%)	9 (2.31%)	HR	1.359	0.615	3.004	0.449

Values are presented as number (%) or incidence per 1000 catheter-days with 95% confidence intervals (CI). Primary outcomes were analysed in both the intention-to-treat (ITT) and per-protocol (PP) populations. Relative risks (RRs) and absolute risk differences (ARDs) with 95% CIs were estimated using generalized estimating equation models with robust standard errors to account for ward-level clustering, with study period included as a fixed effect. Incidence rates per 1000 catheter-days and their 95% CIs were calculated using exact Poisson methods based on the first occurrence of phlebitis per patient. Hazard ratios (HRs) were estimated using Cox proportional hazards models. ARD is presented as percentage points for non-inferiority assessment. CRBSI effect estimates were not calculated because no events occurred in either group. All analyses were conducted at the patient level. Abbreviations: ARD, absolute risk difference; CI, confidence interval; CRBSI, catheter-related bloodstream infection; HR, hazard ratio; ITT, intention to treat; PP, per protocol; RR, relative risk.

**Table 4:** Comparison of resource use and peripheral intravenous catheter dwelling time by treatment group.

Outcome	Clinically Indicated	Routine Replacement	Difference (95% CI)			*p*
	(*n* = 656)	(*n* = 629)	Estimate	Lower	Upper	
	X ± SD	X ± SD				
	Median [Range]	Median [Range]				
Duration of IV therapy (h)	169.19 (230.04)	167.67 (251.54)	13.34	−24.65	27.7	0.910
Total PIVC used	1.77 (1.34)	2.16 (1.44)	−0.386	−0.539	−0.235	<0.001
	1 [1–14]	2 [1–10]				
PIVC dwell time (h)	111.98 (32.47)	89.57 (6.30)	22.409	19.885	24.933	<0.001
	104.0 [5.4–350.0]	89.1 [14.3–120.4]				

Values are presented as mean ± standard deviation (SD). Medians and ranges are additionally presented for total PIVC use and PIVC dwell time. Differences are presented as mean differences with 95% confidence intervals (CI). Analyses were conducted using the per-protocol population at the patient level. *p*-values were calculated using the independent *t*-test. Comparison of resource use and PIVC dwell time by treatment group. Abbreviations: CI, confidence interval; IV, intravenous; PIVC, peripheral intravenous catheter; SD, standard deviation.

## Data Availability

The data that support the findings of this study are not publicly available due to institutional and patient privacy restrictions but are available from the corresponding author, J.K., upon reasonable request.

## Abbreviations

The following abbreviations are used in this manuscript:

ARD	Absolute risk difference
CI	Confidence interval
CRBSI	Catheter-related bloodstream infection
CRIS	Linear dichroism
CRXO	Cluster-randomised crossover
GEE	Generalized estimating equation
HR	Hazard ratio
ICC	Intraclass correlation coefficient
INS	Infusion Nurses Society
ITT	Intention to treat
IV	Intravenous
PIVC	Peripheral intravenous catheter
PP	Per-protocol
RR	Relative risk
SD	Standard deviation
